# The Use of Vacuum Closure-assisted Device in the Management of Compound Lower Limb Fractures with Massive Soft Tissue Damage

**DOI:** 10.7759/cureus.5104

**Published:** 2019-07-09

**Authors:** Stavros Angelis, Alexandros P Apostolopoulos, Lefteris Kosmas, Theodore Balfousias, Athanasios Papanikolaou

**Affiliations:** 1 Orthopaedics, General Hospital Hellenic Red Cross Korgialenio - Benakio, Athens, GRC; 2 Orthopaedics, East Surrey Hospital, Surrey and Sussex Healthcare National Health Service Trust, Redhill, GBR

**Keywords:** vacuum-assisted closure device, negative pressure wound treatment, compound fracture, wound debridement, flap

## Abstract

The simultaneous exposure of tissue and bone poses specific management challenges. Patients with extended soft tissue damage and high-grade compound fractures present a demanding clinical challenge, requiring a complex approach and multiple orthopaedic, plastic, and vascular-reconstructive procedures. Management involves combinations of wound debridement and closure by secondary intention, use of vacuum-assisted closure (VAC) devices, and various reconstructive plastic surgery methods. We present three consecutive complicated cases, involving compound fractures of the lower limb with massive soft tissue damage (Gustilo-Anderson type IIIB) that were managed with debridement, application of external fixation and VAC device. The mean wound size was 24 cm in length and 12 cm in width. The aim of treatment was to cover the bone with soft tissue and achieve healing of the fracture without persistent infection. Wound healing was achieved in all three cases within 30-42 days (mean 34). In one case, the skin graft was applied on day 33.

Utilizing this method as part of a multi-directional approach, the VAC system helps the patient recover faster. Moreover, it acts as a feasible and valuable method to treat compound fractures with massive soft-tissue defects. VAC can replace microsurgical soft-tissue transfer, reduce the risk of infection and allow salvaging the limb.

## Introduction

Fractures accompanied by an open wound, at or near the fracture site, are called open or compound [1]. The simultaneous exposure of tissue and bone poses specific management challenges. As with most wounds, damage to the soft tissue increases the risk of infection [1-3]. However, prophylaxis against osteomyelitis is also a key factor in treatment. The severity of open fractures is generally assessed using the Gustilo-Anderson open fracture classification system [1]. This classification system evaluates the injury severeness according to wound size, contamination and tissue damage.

Three consecutive cases of compound lower limb fractures with extensive soft tissue damage (Gustilo - Anderson IIIB) are presented. Management approach with the use of external fixation, debridement, and vacuum-assisted closure (VAC) device is demonstrated. Afterward, discussion upon current concepts around wound management is attempted.

## Case presentation

Case 1

**Figure 1:**
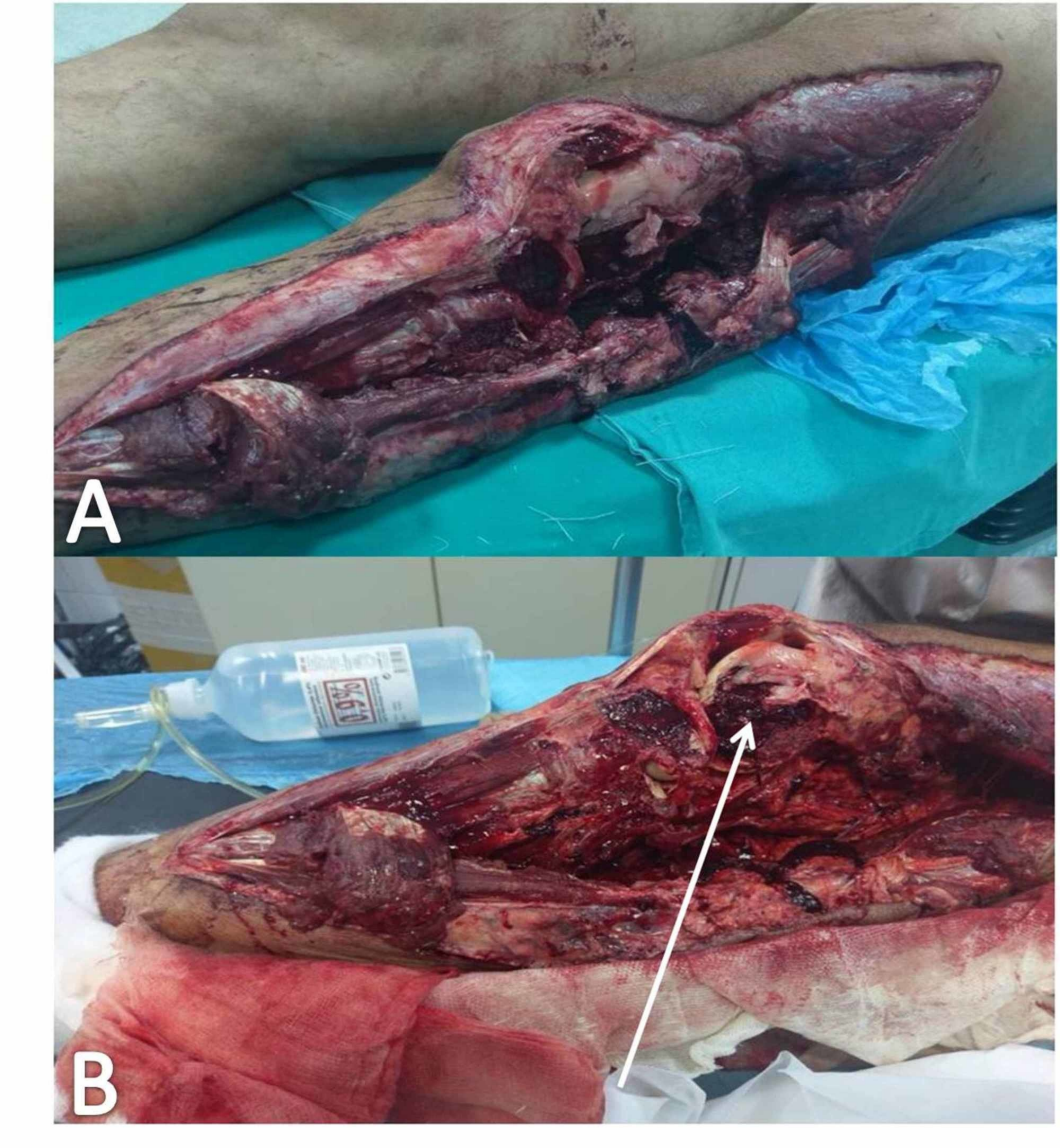
Massive soft tissue damage at the lateral side of the left femur and tibia The wound on arrival at the accident and emergency Department (A); compound fracture (B) of the lateral femoral condyle (white arrow)

A 56-year-old man with a massive soft tissue lesion at the lateral side of the left femur and tibia was transferred to the Accident and Emergency Department (AED) after a road traffic accident. The patient was resuscitated according to the Advanced Trauma Life Support (ATLS) protocol. After stabilization of his vital signs, an imaging study was performed. X-rays and computed tomography (CT) scan revealed a fracture of the lateral femoral condyle and a comminuted fracture of the proximal third of the left tibia (Gustilo-Anderson type IIIB). These fractures were associated with extended soft tissue damage (wound defect size 37 cm x 15 cm, 555 cm²) at the lateral side of the left femur and tibia (Figures 1A, 1B). A cast was placed for provisional stabilization. The primary and secondary survey did not reveal any other major injury except mild concussion and peri-traumatic amnesia.

**Figure 2:**
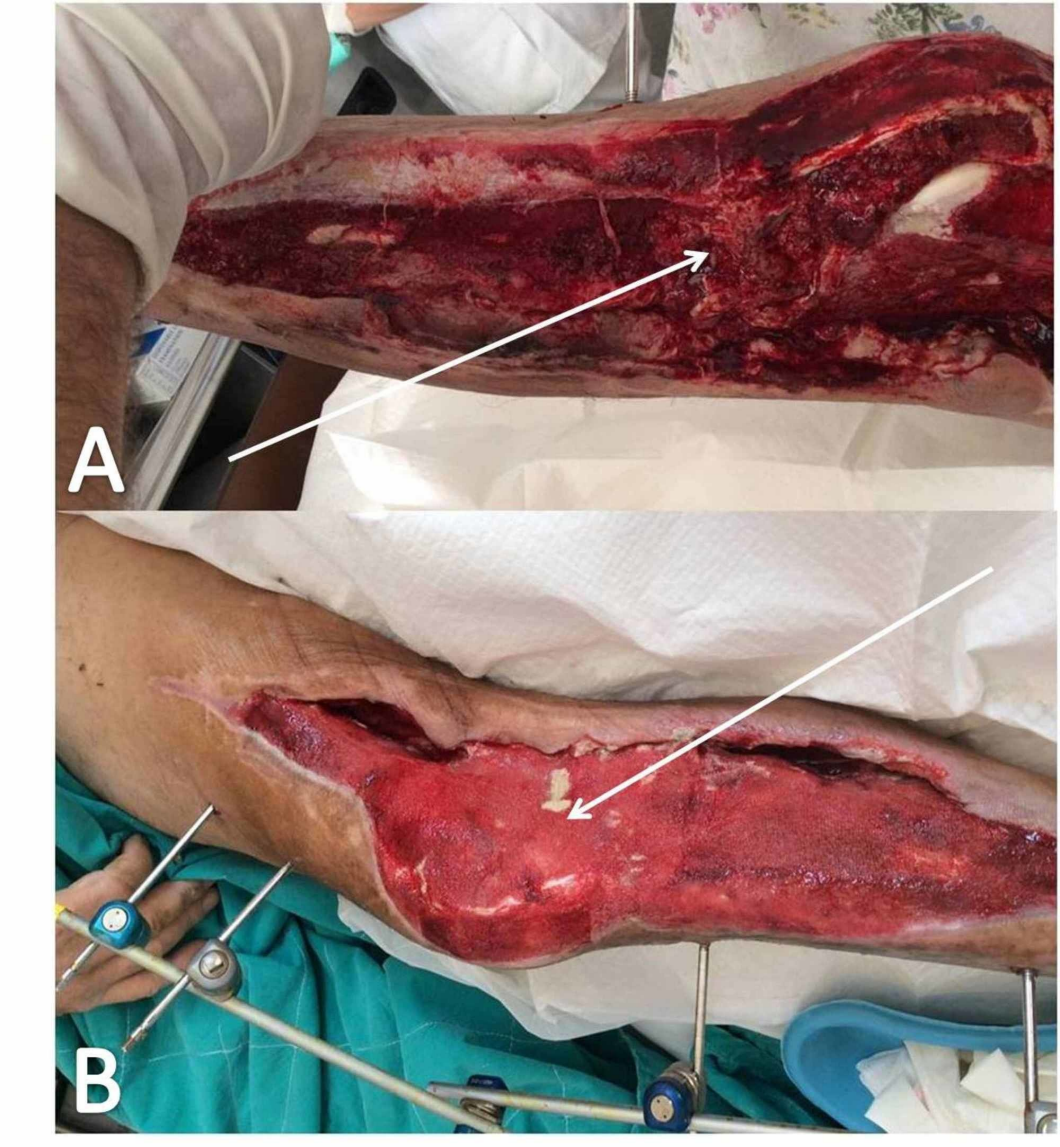
Wound following NPWTS treatment On day 26 (A); on day 30 (B) NPWTS, negative pressure wound therapy system

**Figure 3:**
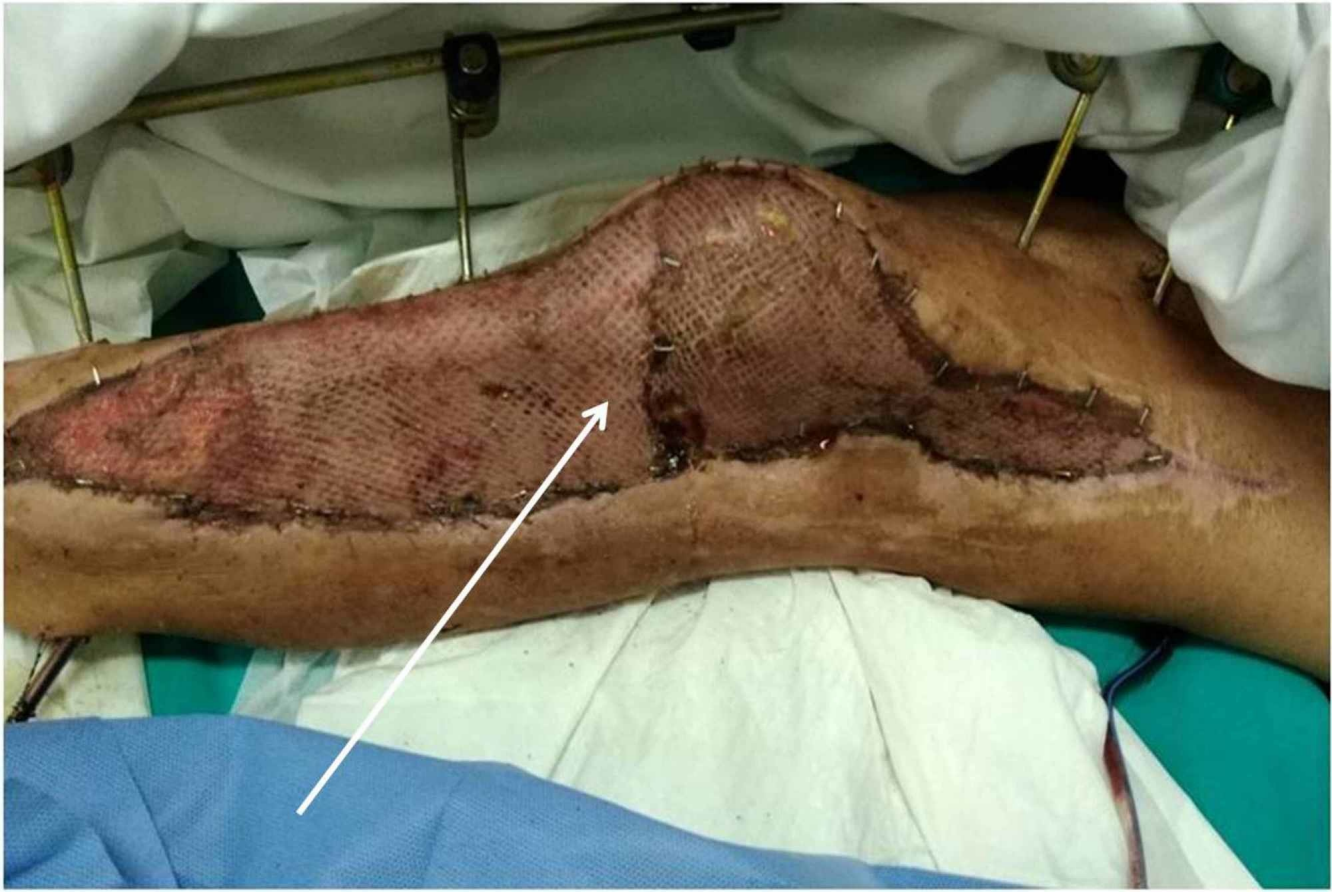
First wound change five days after the application of skin graft

The patient was taken to the theatre. Debridement and exploration of the wound were performed. The fracture was reduced and immobilized using an external fixation device (Hoffmann® II External Fixation System Stryker®). Forty-eight hours post-injury, the patient was taken back to the theatre. Debridement of the wound and a negative pressure wound therapy system (NPWTS) was applied (pressure applied on 125mmHg - Simex 300 ®). The NPWTS was replaced every four days (Figures 2A, 2B). On day 33, the patient was taken to the theatre for a final review and an autologous skin graft was used to cover the skin defect (Figure 3). The skin graft was harvested by the anterior aspect of the femur. The patient was discharged on day 40 and was followed up on a regular basis at the outpatients' department.

Case 2

**Figure 4:**
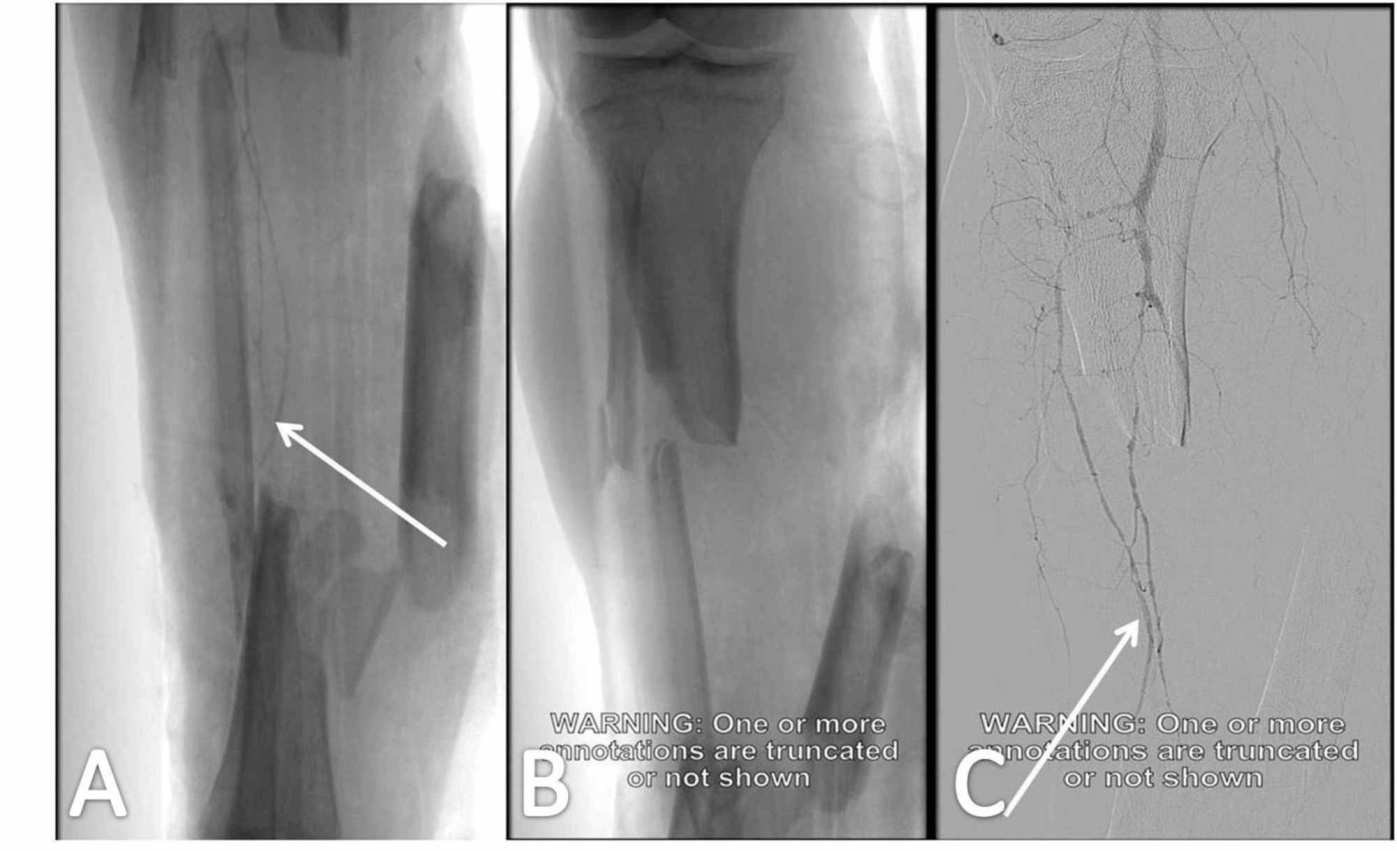
Vascular investigation with CT angiography Patent vascular network of the lower limb (white arrows in Figures A and C); comminuted and segmental fracture of the right tibial and fibular shaft (A, B) CT, computed tomography

A 35-year-old male patient was transferred to the AED due to a motorcycle road traffic accident. ATLS protocol was applied and the patient's vital signs were stabilized. The primary survey revealed a comminuted and segmental fracture of the right tibial and fibular shaft with a 22 x 11-cm wound (222 cm²) defect on the posterior, medial and frontal aspect of the distal third of the tibia. Vascular investigation (CT angiography) revealed patency of the vascular network to the lower limb (Gustilo-Anderson Type IIIB open fracture; Figures 4A-4C). Provisional stabilization was achieved with a simple cast. No other important injury was noticed.

**Figure 5:**
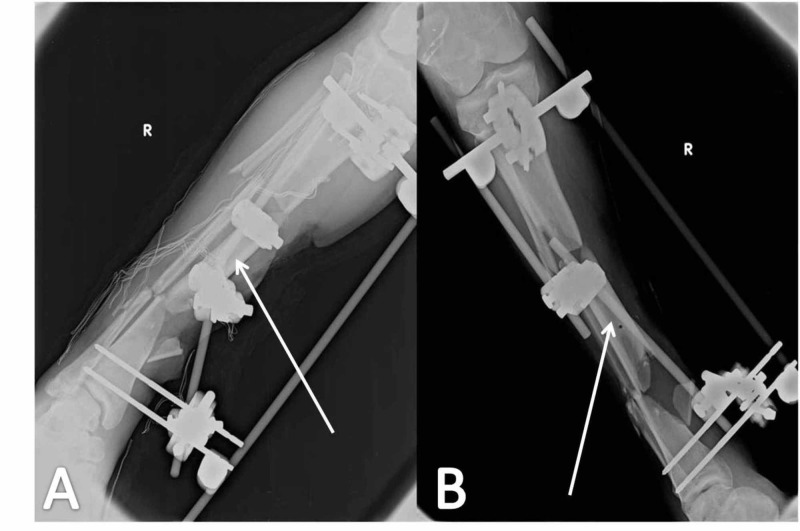
Application of external fixation White arrows (A, B) point to the loose intermediate bone section of the tibial shaft.

**Figure 6:**
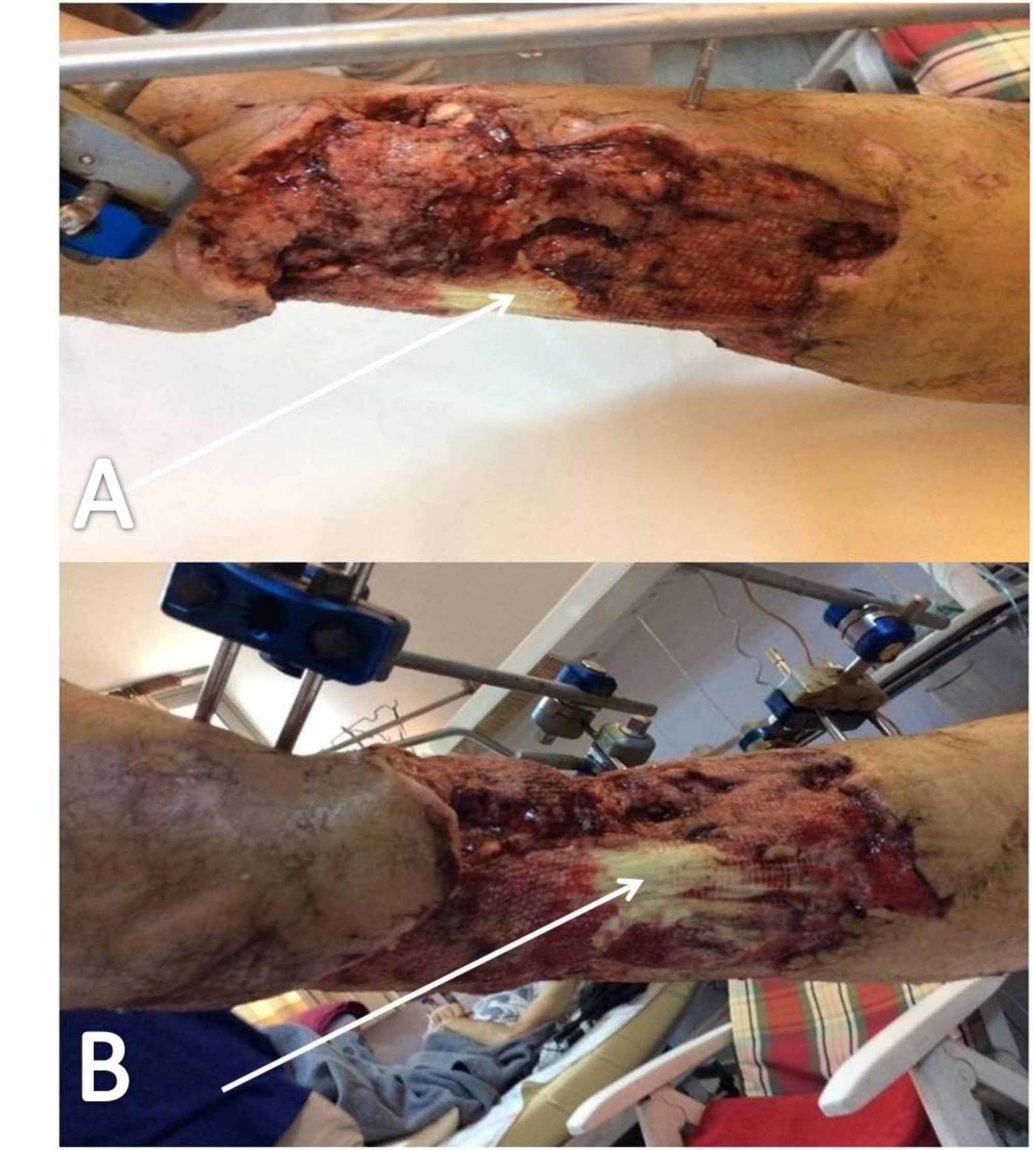
Wound appearance one week postoperatively, after radical surgical debridement and NPWTS application White arrows (A, B) point to the exposed tibialis anterior tendon. NPWTS, negative pressure wound therapy system

**Figure 7:**
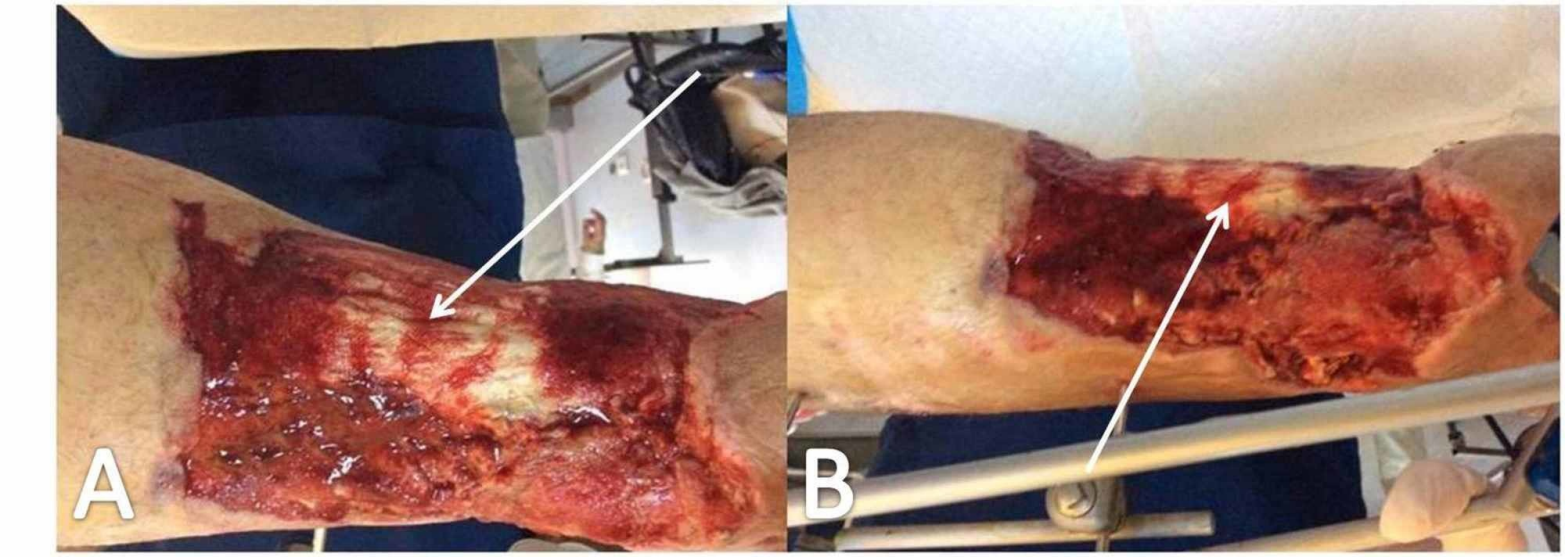
Wound appearance two weeks postoperatively White arrows (A, B) point to signs of tissue regeneration over the exposed tibialis anterior tendon.

**Figure 8:**
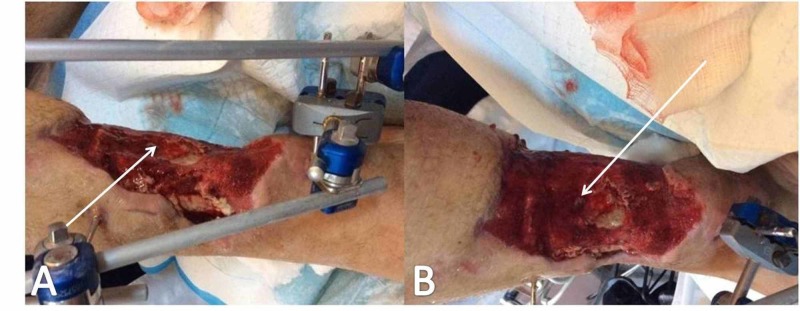
Three weeks following NPWTS application in a four-day interval Arrows point to impressive tissue regeneration over tibialis anterior tendon; white arrows (A, B) point to impressive tissue regeneration over the tibialis anterior tendon. NPWTS, negative pressure wound therapy system

The patient was admitted and transferred to the operation room where the wound was explored and debrided and an external fixation system was applied (Hoffmann® II External Fixation System Stryker®; Figures 5A, 5B). Forty-eight hours post-injury. the patient was taken back to the theatre and an NPWTS was applied (pressure applied on 125mmHg - Simex 300 ®). The postoperative treatment included a VAC device change every four days (Figures 6-8). The patient was discharged on day 35 and was followed up in the orthopaedics outpatients' department on a regular basis.

Case 3

**Figure 9:**
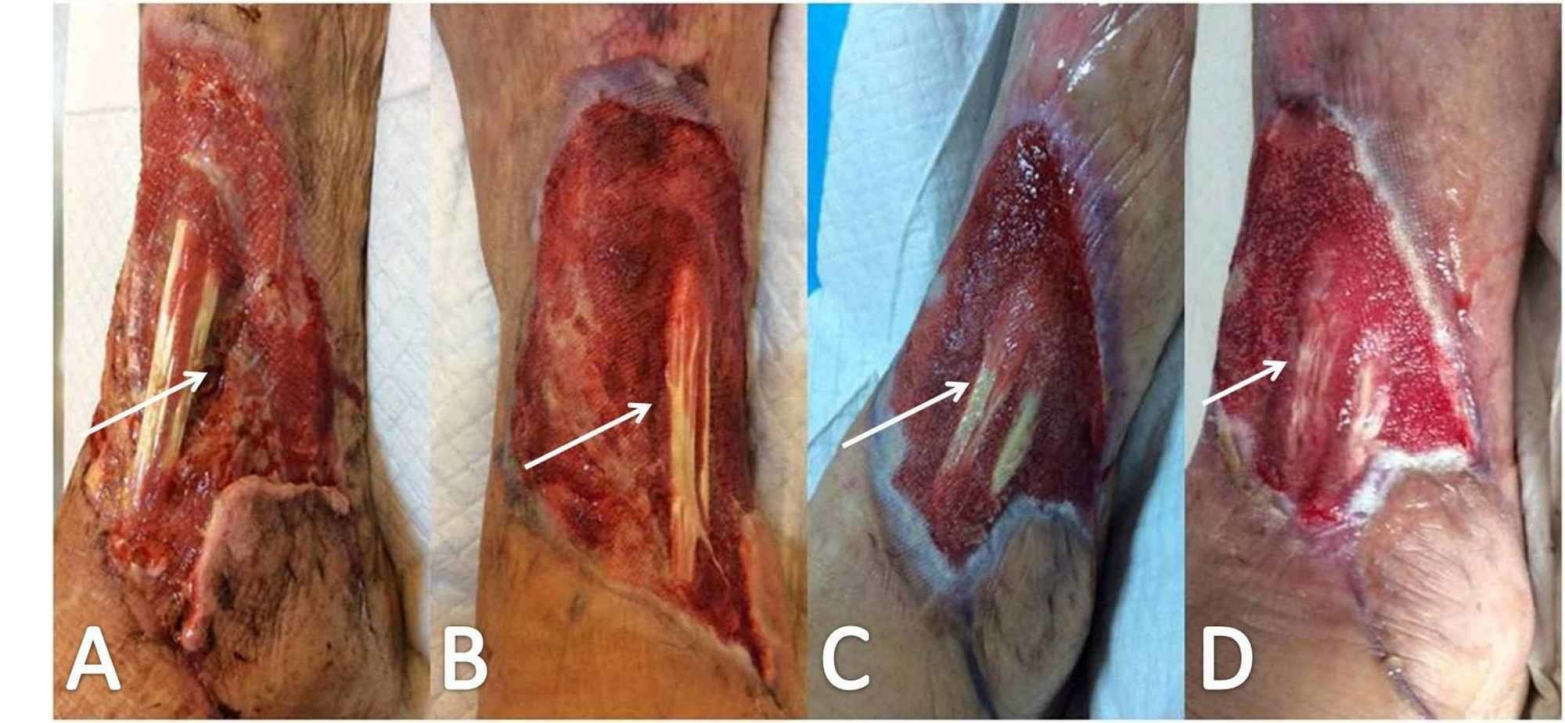
Gradual wound healing process Notice tissue regeneration and progressive filling of tissue gap through Figures A to D (white arrows).

An 80-year-old male patient was transferred to the AED due to an accident with an agricultural machine. ATLS protocol was applied and the patient's vital signs were stabilized. The primary survey revealed a compound comminuted fracture of the left tibial shaft with a 15 x 12-cm (180 cm²) wound defect on the anterior and lateral aspect of the distal third of the tibia. CT angiography did not reveal any vascular damage (Gustilo-Anderson type IIIB open fracture). Provisional stabilization was achieved with a simple cast. No other important injury was noticed. The patient was admitted and transferred to the operation room where the wound was explored, debrided and an external fixation system was applied (Hoffmann® II External Fixation System Stryker®). Forty-eight hours post-injury, the wound was secondly explored and debrided and an NPWTS system was applied (pressure applied on 125mmHg - Simex 300 ®). Postoperative treatment included VAC change every four days (Figure 9). On day 28, the patient was discharged. The wound healed by secondary intention. The patient was followed up in the orthopaedics outpatients' department on a regular basis.

## Discussion

Management of compound fractures with extensive soft tissue damage is a challenging task for the trauma and orthopaedic team. Patients are usually with polytrauma and have suffered multiple injuries. A multidisciplinary approach is always necessary for managing these patients. Damage control principles are imperative for the initial management of the fracture and the soft tissue lesion. Stabilization of the fractures, wound debridement and exploration are the initial actions that should be applied the soonest possible. Delayed primary wound closure by multiple surgical debridements, application of negative wound pressure (NWP) devices or even early flap coverage are acceptable options alone or in combination for final treatment. The aim in the management of open fractures is early stabilization and achievement of soft tissue coverage before the infection develops. It has been estimated that 27% of these wounds become infected and therefore thorough management is required in order to prevent complications such as non-union, chronic osteomyelitis, morbidity, and even amputation [1-2].

Wound management with an NWP device involves the application of an airtight wound dressing through which negative pressure facilitates the wound and tissue fluid to be drawn and collected into a canister. In our cases, the NPWTS was applied after re-evaluating the wound and after the debridement had been performed (48-52 hours post-injury). The wound dressing was replaced every four days. Kim et al. have suggested that considering patient comfort, the costs related to the NPWTS, and the final flap results, a seven-day interval between changes of the NPWTS is acceptable [2].

NPWTS is acting via several different mechanisms. The negative pressure causes deformation of the wound, bringing the skin edges closer together and therefore reducing the volume of tissue and skin needed to heal the wound [1]. Moreover, the increase in the capillary flow which is caused by the tension across the tissue stimulates granulation tissue formation [4]. Subsequently, the removal of wound exudate and its enzymes prevents further tissue damage and reduces the skin dressing changes, keeping the surrounding skin dry [1]. VAC seals the wound with a foam dressing and applies negative pressure to the wound bed. The removal of exudate and infectious material supports the cleaning of the wound [3]. Together with the reduction of oedema and the increase in blood flow, this promotes the formation of granulation tissue and eventually wound closure by a split skin graft.

The use of NPWTS has been widely increased during the last decade. It was estimated that in the United States of America, the Medicare payments for NPWTS increased from 24 million USD (United States Dollars) in 2001 to 164 million USD in 2007 [5]. However, is this increased use of NPWT justified? Liu et al. conducted a systematic review in order to clarify the detailed advantages and disadvantages of the negative pressure wound therapy (NPWT) in the treatment of open fractures in comparison to the conventional wound dressings [6]. In the eight randomized controlled trials (421 patients) and the six retrospective cohort studies (488 patients), that were reviewed, NPWTS resulted in a significantly lower infection rate, shorter wound coverage time, shorter healing time and hospital stay length, and lower amputation rates. However, no statistically significant difference was found in the need for flap surgery.

Similarly, Kricka et al. concluded that the use of NPWTS in managing grade III compound fractures statistically reduced the bacterial contamination at the site of injury and also reduced the incidence of infectious complications [7]. Stannard et al. added that NPWTS increases the “take rate” of skin grafts, skin substitutes, and composite skin grafts and allows quicker graft incorporation [8]. In regards to fracture healing, there is no data supporting the use of NPWTS. Cohort studies have failed to find any advantage or disadvantage of NPWTS compared to the conventional wound dressing, and this might be due to the preoperative intergroup incomparability or the inadequate sample size.

There are only a few recent studies that have questioned the use of NPWTS in the management of compound fractures. Cook et al. have suggested that there is no benefit in using NPWTS over standard dressings [9]. Moreover, Costa et al. published the results of a multicentre randomized controlled study that compared 460 patients in total, that had suffered compound lower limb fractures and had received NPWTS (226) and standard dressing treatment (234) [10]. The investigators found no difference in the Disability Rating Index within 12 months. Moreover, there was no difference found in the number of surgical site infections within 12 months, suggesting overall that NPWTS dressings do not provide a clinical or an economic benefit for patients with an open fracture of the lower limb. These studies, however, do not clarify whether the investigators took into consideration the difference in the outcomes in regards to the size and the extent of the soft tissue defect. Our cases presented with massive soft tissue defects measuring from 180 cm² to 555 cm² (mean 319 cm²). It is our strong belief that conventional wound dressing change would prolong the healing time and increase the infection rate. Moreover, conventional wound dressing change which is usually required every two days is not easily tolerated by the patients who have massive soft tissue damage, and general anaesthesia is often required due to the great discomfort and pain. The NPWTS dressings were replaced every four days in our series, prolonging in that way the dressing interval and reducing cost and discomfort (Table 1).

**Table 1:** Treatment characteristics in our case series NPWTS, negative pressure wound therapy system

Hospitalization	28-40 days (mean 34.6 days)
Mean Flap Size	319 cm²
Mean Healing Period	34 days
Number of NPWTS Changes	6-9 (mean 7)
NPWTS-related cost	1100-1320 € (mean 1.210 €)

## Conclusions

Utilizing the method of NPWT as part of a multi-directional approach helps the patient recover faster. It acts as a feasible and valuable method to treat compound fractures with massive soft-tissue defects that can replace microsurgical soft-tissue transfer, and also reduces the risk of infection and allows salvaging of the limb. It is also our strong belief that the vacuum-assisted closure system compared to the conventional wound dressing change prolongs the dressing intervals, reduces cost, healing time, and discomfort to the patient.
